# Malassezia (Pityrosporum) Folliculitis Masquerading As Recalcitrant Acne

**DOI:** 10.7759/cureus.13534

**Published:** 2021-02-24

**Authors:** Vikas Malgotra, Harjap Singh

**Affiliations:** 1 Dermatology, Government Medical College, Jammu, IND; 2 Internal Medicine, Narayana Superspeciality Hospital, Katra, IND

**Keywords:** steroid acne, malassezia folliculitis, pityrosporum folliculitis, recalcitrant acne, fungal acne, itchy red pimples on face, malassezia furfur, pityrosporum ovale

## Abstract

Malassezia (Pityrosporum) folliculitis is a relatively common skin infection that affects the hair follicles. The condition is characterized by monomorphic perifollicular skin lesions and itching without comedones. Malassezia folliculitis significantly resembles acne vulgaris and steroid acne but is subtly distinct and managed differently. Oral antifungals are preferred for the treatment and result in a dramatic improvement in the disease condition. Early recognition of the disease is important for satisfactory clinical outcomes. This case reports about a female in the reproductive age group, who took multiple treatments for erythematous papular lesions on her face with a provisional diagnosis of acne vulgaris. After observing no improvement over the last three months, she visited the Dermatology clinic at a tertiary care hospital. A diagnosis of Malassezia folliculitis was considered and confirmed on microscopic examination and oral and topical antifungals were prescribed. She reported significant improvement in her skin lesions after two weeks of treatment.

## Introduction

Malassezia furfur is a commensal yeast that resides on the skin in normal individuals. It was first identified more than 150 years ago, as a constituent of the commensal flora of the skin. The fungus is a lipophilic, dimorphic basidiomycete. In the presence of certain risk factors, such as immunosuppression or antibiotic intake, the organism can overgrow and give rise to infections. These well-recognized infections include - Pityriasis versicolor and Malassezia folliculitis. In addition, the fungus can interact with the immune system and cause exacerbations of seborrheic dermatitis, atopic eczema, and psoriasis. It is also associated with confluent reticulated papillomatosis, onychomycosis and transient acantholytic dermatosis [1].

Malassezia folliculitis presents as monomorphic papulopustular skin lesions commonly on the trunk, upper arms, and face. Itching is associated with the lesions in around 80% of cases [2]. The condition clinically resembles, commonly misdiagnosed, and can co-exist with acne vulgaris. Antifungal medications result in dramatic clinical improvement of the disease. Regular follow-ups to monitor clinical improvement and patient compliance with treatment are essential.

## Case presentation

**Figure 1:**
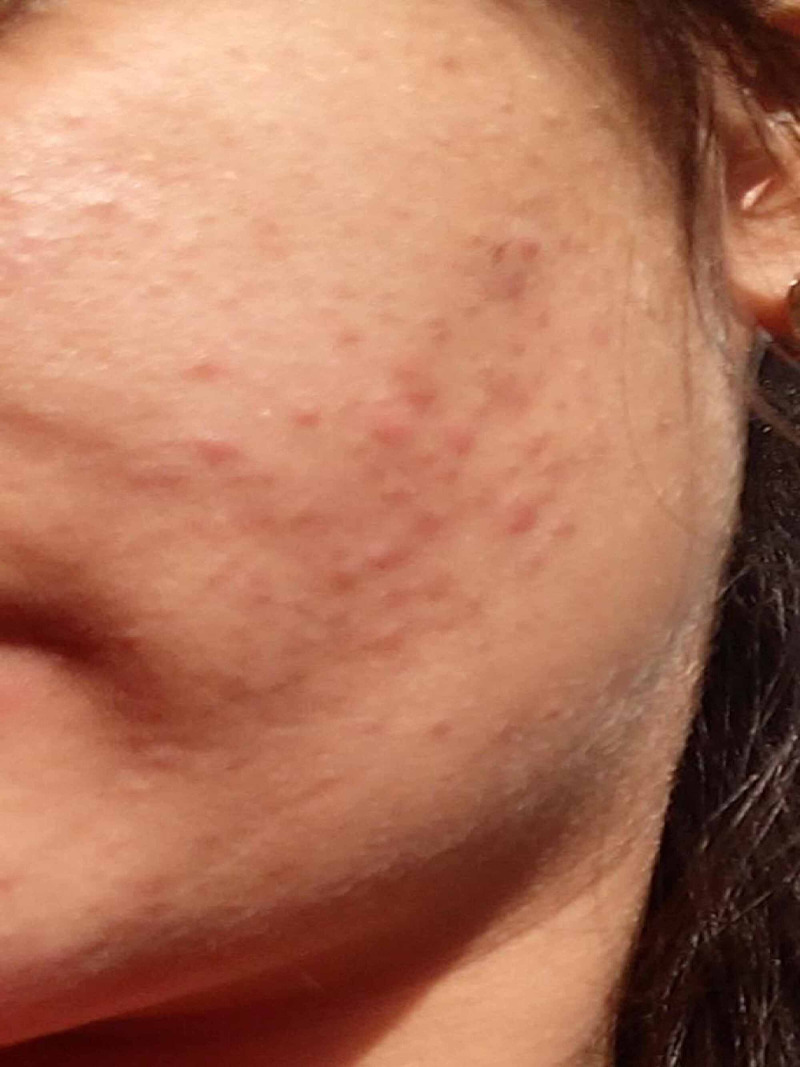
Erythematous papules on the left cheek with no complaints of itching, burning or pain on presentation Small red papules can be noticed on the face with virtually no comedones. Some residual hyperpigmentation can also be noticed in the vicinity, possibly as sequelae of previously resolved acne.

A 24-year-old married female presented in the Dermatology Department at a tertiary care hospital with complaints of pimples on her face, which would not go away even after taking multiple treatments for the last three months including topical clindamycin and nicotinamide, oral antibiotics, and isotretinoin for a provisional diagnosis of acne vulgaris. On evaluation, she was found to have multiple erythematous papules with few pustules limited to the face involving both cheeks and sparing the skin around the nose, mouth, and over the forehead (Figure 1). She did not report any itching or burning sensation over the lesions and the rest of her body, including the trunk, was not involved.

**Figure 2:**
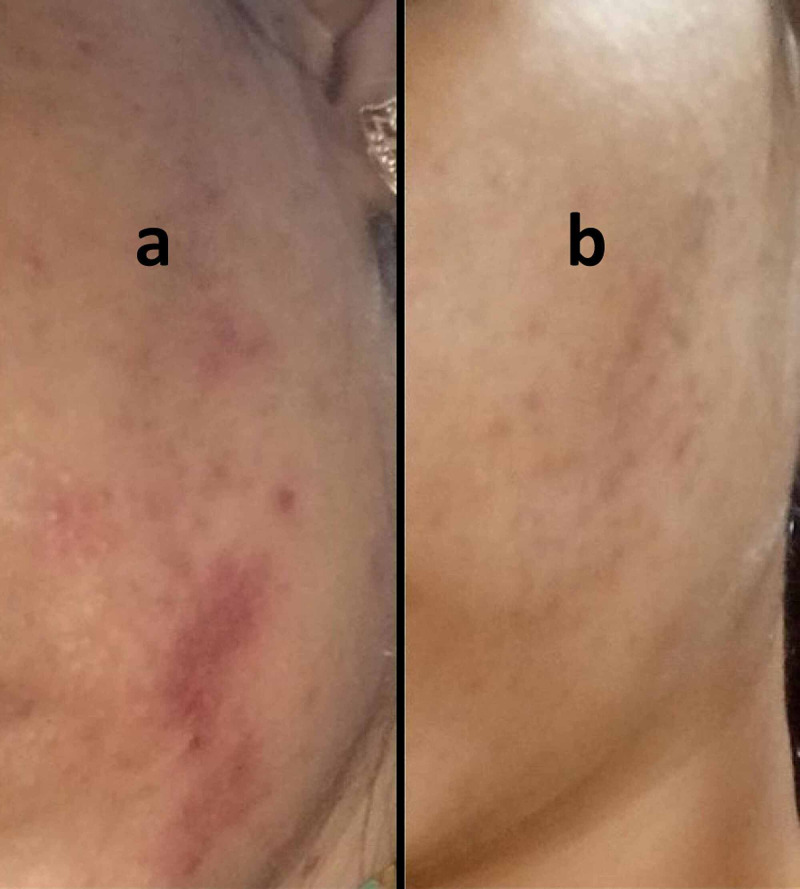
Exacerbation of signs and symptoms after starting medications for acne, and nearly complete resolution after starting antifungals. (a) Increased erythema with burning sensation over underlying lesions on the face reported after topical application of clindamycin-tretinoin and intake of oral antibiotics for a provisional diagnosis of acne vulgaris. (b) Lesions showed significant resolution after a course of topical and oral antifungals.

On further questioning, she reported having excessive facial hair, recent weight gain, irregular menstrual cycles, and also failure to conceive a child despite trying for a couple of months. She was investigated for polycystic ovarian disease (PCOD) and thyroid dysfunction and was started on oral antibiotics, a salicyclic acid facewash, and a formulation containing topical clindamycin and tretinoin. Two days later, the patient reported back to the hospital complaining of excessive burning sensation and erythema around the papules, which she correlated to the application of topical formulation (Figure 2(a)). She was counseled about the possibility of this adverse drug reaction arising due to applying the formulation containing tretinoin. She then asked if it could be discontinued and a combination of clindamycin with nicotinamide was prescribed instead.

The laboratory investigations on this follow-up revealed no alteration in her hormonal profile but ultrasonography findings were suggestive of PCOD. The patient also had an elevated thyroid-stimulating hormone (TSH). She was counseled about the importance of dietary modification and weight loss for the control of PCOD and thyroid dysfunction. After applying the topical medication for two weeks along with oral antibiotics, which she was taking previously for a provisional diagnosis of acne vulgaris, the patient returned for a follow-up visit. The patient was not happy with the treatment and there was no objective improvement in her clinical condition.

A diagnosis of Malassezia (Pityrosporum) folliculitis was now considered in view of the predominant papules and pustules with the lack of comedones over the face. Skin scrapings taken from the papules and examined under the microscope after the addition of potassium hydroxide (KOH) were positive for fungal hyphae and spores. The patient was then started on topical antifungal lotion in addition to the therapy for acne vulgaris. One week later, the patient reported partial improvement. The treatment regimen was revised and two weeks of Itraconazole were prescribed, topical antifungals were continued, and oral antibiotics discontinued. After completing the prescribed treatment for two weeks, the patient reported significant improvement and resolution of nearly 90% of her skin lesions (Figure 2(b)). She was planned to be maintained on oral Itraconazole after gradual dose reduction, but oral antifungals had to be discontinued due to their teratogenic potential as she was trying to conceive while being on treatment. Topical antifungals were prescribed instead, and the patient was counseled on clinical outcomes with follow-up in approximately one month.

## Discussion

Malassezia (Pityrosporum) folliculitis is an infection of the hair follicles caused by the commensal lipophilic yeast, Malassezia furfur. Malassezia is a genus that mostly contains species of lipophilic and non-lipophilic yeasts. These fungi were identified over 150 years ago when the fungus associated with Pityriasis versicolor was recognized to have dimorphic characteristics - both yeasts and filamentous forms. The fungus associated with seborrheic dermatitis was identified as existing in yeast forms around the same period [1]. Over the years, more species were included in the genus. Malassez is credited with the discovery of yeast-like cells in the stratum corneum of the skin in 1874 and the genus was initially named after him. When the organism was cultured later, the name given to the fungus was Pityrosporum ovale. For many years, Pityrosporum and Malassezia were thought to be two distinct genera. A consensus was eventually reached to prefer the name Malassezia over Pityrosporum after revision of taxonomical characteristics of the organisms in the two genera [1,3].

Malassezia folliculitis was first described as an acneiform eruption associated with broad-spectrum antibiotic use by Weary et al. in 1969. The disease is caused by an overgrowth of the fungus present as a part of the commensal skin flora [4,5]. The fungus assumes a pathogenic role if the flora is disturbed due to intake of broad-spectrum antibiotics or steroids/immunosuppression. It mostly causes indolent skin infection but systemic infections are relatively common occurrences in intensive care units.

The skin infection is common in adolescents and adults aged 11-30, with an average age of infection as 26 years. According to a study conducted on 49 patients by Durdu et al., the face and trunk were the most common sites of involvement in more than 50% of cases, with extremities being the other most commonly affected sites [2,6]. The predominant distinguishing features are involvement of the trunk, extremities, and face, as well as the presence of intense itching, and monomorphic papular and pustular skin lesions with a lack of comedones. The epidemiological studies related to the prevalence and incidence of the disease are scarce. There is an increased incidence of infection in hot and humid environments.

The condition needs to be clinically differentiated from acne vulgaris, follicular eczema, bacterial folliculitis, eosinophilic folliculitis, tinea incognito, and steroid acne.

Direct microscopic or histopathological examination of fungal hyphae and spores is helpful in making a diagnosis. Examination of the skin lesions under Wood’s lamp with yellow-green fluorescence can also be suggestive of fungal infection [6].

Lack of response to the empirical treatment for acne vulgaris should prompt the dermatologist and general healthcare practitioners to consider alternate diagnoses, including Malassezia folliculitis for better patient outcomes.

The goal of the treatment is to mitigate the fungus present on the skin and establish a pre-infection commensal relationship. Antifungal medications are not prescribed to eradicate the fungus. Oral antifungal medications are more effective than topical medications for the control of infection. This is perhaps due to better hair follicle penetration [7,8].

## Conclusions

Malassezia folliculitis is an underdiagnosed clinical condition that requires timely and accurate diagnosis and management. This can significantly reduce the patient discomfort and cost of treatment. The condition requires a high index of clinical suspicion on initial presentation, especially if there is intense itching and monomorphic skin lesions are present over the trunk, extremities, and face. Alternate clinical diagnoses should also be considered if inadequate clinical improvement or worsening of the disease occurs with a properly planned treatment regimen for acne. Administration of antifungal medications results in a dramatic improvement of the disease.
